# Supplementary material for: Effect of Mechanical Damage in Green-Making Process on Aroma of Rougui Tea
Source: Foods. 2024 Apr 25;13(9):1315. doi: 10.3390/foods13091315 (PMC11083345; doi:10.3390/foods13091315)
Supplement: Supplementary file 1 [file foods-13-01315-s001.zip › Table S1.pdf]

Table S1 q-PCR reaction system

reagent	capacity
Template	2 μL
Forward Primer	0.4 μL
Reverse Primer	0.4 μL
2×Perfect StartTM Green qPCR SuperMix	10 μL
Dye（II)	0.4 μL
ddH_2O	6.8 μL
