# Supplementary material for: Effect of Mechanical Damage in Green-Making Process on Aroma of Rougui Tea
Source: Foods. 2024 Apr 25;13(9):1315. doi: 10.3390/foods13091315 (PMC11083345; doi:10.3390/foods13091315)
Supplement: Supplementary file 1 [file foods-13-01315-s001.zip › Table S2 Real.pdf]

Table S2 Real-time PCR gene primersequence

Gene name	Gene ID	Primers (5'-3')
CsAFS	TEA031095.1-qpcrF	CACACCAACCCTAGACGAGTA
	TEA031095.1-qpcrR	CGCAGAAGTTCCAAGGTCATT
CsAFS	TEA009166.1-qpcrF	GAGGAGATAGCCAGGAAGAAGAT
	TEA009166.1-qpcrR	GCCACTCTAGCTGTGTTGAC
CsHMGS	TEA014739.1-qpcrF	GCCAGCGAATAGTAATGTTCTCA
	TEA014739.1-qpcrR	ACCTATGCTCCATCACCTTCA
CsLOX	TEA011765.1-qpcrF	GCGTGACAGAGCCTTACATAA
	TEA011765.1-qpcrR	AGAAGCAAGTCTCAATGATTCCA
CsACAA	TEA015906.1-qpcrF	GCAATTCCAGCAGCAGTGAA
	TEA015906.1-qpcrR	GCCGCCATTGACATTGACTT
CsMEP	TEA027322.1-qpcrF	GGCTGATACTGTCGGTCCTAA
	TEA027322.1-qpcrR	CCTTTCGTTGCTCTTTCTTCCA
CsACX	TEA019216.1-qpcrF	CTCTTCCTTCTTCACAAGCATCAA
	TEA019216.1-qpcrR	TTCCATCATAGCAGCCGAGTA
CsISPG	TEA013763.1-qpcrF	GAGAACTTCCACCAGCAGATG
	TEA013763.1-qpcrR	AGCACCAGATGATAATTCCTTGAG
CsPAL	TEA003374.1-qpcrF	CCGAGCAACACAACCAAGAT
	TEA003374.1-qpcrR	CTTCCAAGTGCCTCAAGTCTATG
CsPAT	TEA033170.1-qpcrF	GGAGCGACGAGATTATTTGGTTA
	TEA033170.1-qpcrR	ATAGCGGCATAAGGACTCTGA
CsOPR	TEA029800.1-qpcrF	GTCTTAGGCTCAAGGCTCAC
	TEA029800.1-qpcrR	TGGACTCCTCATCTTCACTACC
CsASP	TEA024318.1-qpcrF	TTGCTGATGGTGGTGAATGC
	TEA024318.1-qpcrR	AACTGGCTCTCAACTCTGCTA
internal control	*CsGAPDH*-F	TTTTTGGCCTTAGGAACCCAGAGG
	CsGAPDH-R	GGGCAGCAGCCTTATCCTTATCAGT
