# Supplementary material for: Effect of Mechanical Damage in Green-Making Process on Aroma of Rougui Tea
Source: Foods. 2024 Apr 25;13(9):1315. doi: 10.3390/foods13091315 (PMC11083345; doi:10.3390/foods13091315)
Supplement: Supplementary file 1 [file foods-13-01315-s001.zip › Table S3.pdf]

Table S3 The aroma components with different contents of RGT samples

No.	Compounds	Odour description*	RI	Peak area(mean±SD)*10^4				
				SY	CK7	LY7	DM	ZM
1	nerolidol	Floral, green, citrus, waxy	1569	397.02±70.79e	8458.35±511.82b	9762.44±124.96a	3385.72±275.43d	6099.16±214.06c
2	Linalool	Floral, fruity	1101	2604.70±143.52b	3303.35±87.61a	1972.66±13.12c	415.02±37.68e	613.02±48.10d
3	Linalool oxide I	Sweet, floral,creamy	1071	430.50±61.80d	2780.93±218.43a	2313.99±145.02b	579.45±82.95d	758.58±57.34c
4	Linalool oxide Ⅲ	Sweet woody	1170	69.24±5.56c	582.78±16.39a	590.69±40.63a	268.85±35.59b	281.35±21.81b
5	3,7-dimethyl-1,5,7-Octatrien-3-ol	Tropical	1105	213.80±43.83d	734.35±38.71b	549.60±24.17c	920.22±105.34a	787.98±62.34b
6	Phenylethyl Alcohol	Floral, rose-like	1111	155.45±13.28e	517.80±54.34c	862.73±39.95a	307.02±32.66d	613.74±47.50b
7	Benzyl alcohol	Floral, rose-like	1033	104.13±5.22d	182.96±6.81c	538.79±15.90a	146.50±29.61c	406.56±31.22b
8	1-Hexanol	Yeast aroma, Green	867	60.81±9.91c	313.86±9.20b	806.78±101.79a	7.75±1.53d	57.02±4.66c
9	α-Farnesene	Floral	1508	265.71±30.25c	4364.54±197.12a	2993.17±35.56b	14.70±5.33d	21.90±1.41d
10	(E)-β-Famesene	woody	1454	33.56±5.28e	385.83±36.80b	438.92±20.65a	167.93±9.72d	303.35±7.04c
11	(Z)-2-Penten-1-ol	Fruity	760	20.95±3.91e	34.60±1.61d	47.60±4.60c	102.72±16.79b	161.27±9.55a
12	(E)-2-Hexen-1-ol	Herbaceous, green	863	37.09±7.81c	285.56±6.73b	504.92±84.79a	6.34±0.94d	40.51±3.02c
13	(E)-3-Hexen-1-ol	Green, moss, fresh	852	1323.08±143.98a	1475.27±59.02a	1391.53±119.09a	37.21±6.43c	149.34±11.44b
14	5-methyl-2-(1-methylethenyl)-4-Hexen-1-ol	Floral	1253	361.34±4.60e	709.05±29.35d	4013.62±173.37a	988.03±102.46c	2328.32±121.76b

15	Indole	Floral, animal-like	1291	235.37±33.44e	4989.99±176.95b	5341.94±152.03a	1225.25±99.60d	1803.48±91.38c
16	Phenylacetonitrile	Aromatic	1135	34.81±5.94d	1166.52±150.63a	843.38±22.08b	228.96±20.11c	286.86±19.29c
17	Benzaldehyde	Honey, floral	958	13.87±3.86d	38.42±3.29c	66.59±1.57b	43.03±5.35c	122.83±8.42a
18	Phenylacetaldehyde	Honey-like, sweet	1041	3.57±1.20d	90.60±16.29b	129.30±5.51a	47.87±5.67c	115.68±7.51a
19	Citral	Lemony	1268	5.46±1.50d	7.78±1.23d	52.12±3.30a	16.49±2.75c	31.63±2.70b
20	β-Cyclocitral	Sweet, herbal, hay-like	1217	20.76±3.35c	7.47±0.89d	16.12±0.99c	54.30±6.36b	79.83±6.10a
21	(E)- 2-Hexenal	Grassy green	850	0.00	0.00	24.49±7.40a	4.01±0.47c	14.95±1.56b
22	Jasmone	Herbal, floral, spicy, celery	1392	118.48±11.36e	853.59±43.58b	1104.85±4.77a	329.80±37.30d	486.74±22.69c
23	(E)-α-Ionone	Floral, woody, violet-like	1426	19.36±1.84d	17.86±0.67d	40.95±0.50c	173.16±24.82b	363.95±26.48a
24	β-Ionone epoxide	Fruity	1480	5.72±0.54c	5.38±0.19c	12.21±0.42c	111.20±19.26b	273.35±18.24a
25	Jasmine lactone	Jasmine-like, floral Sweet	1489	217.44±27.09e	5099.79±334.12b	7129.95±144.25a	1829.08±222.08d	3382.43±101.51c
26	Methyl salicylate	Fresh, faint gingery, grass	1190	796.31±56.92c	1127.21±103.86b	1374.89±16.27a	174.98±26.81e	381.65±30.76d
27	2,6-Octadienoic acid, 3,7-dimethyl-, methyl ester	Flowery and honey aroma	1321	20.45±3.55c	16.05±0.33d	43.09±1.41a	10.77±1.91e	30.12±2.47b
28	1-Hexyl acetate	Fruity	1013	105.19±13.09c	388.76±23.35b	1150.63±61.86a	6.94±0.41d	106.90±9.51c
29	Hexyl hexanoate	Fruity odour	1381	95.38±4.53e	239.02±38.83c	882.52±48.82a	182.27±15.54d	452.33±35.75b
30	(E)-3-Hexenyl butyrate	Fruity odour	1212	930.12±59.05c	1602.06±113.45b	2365.09±59.18a	92.26±9.69e	256.30±20.93d
31	Acetic acid, phenylmethyl ester	Honey	1161	8.87±0.97c	8.39±0.28c	22.76±0.39a	6.79±0.25c	14.57±0.58b
32	Nonanoic acid, methyl ester	Fruity	1925	4.15±0.66d	7.40±0.32d	185.00±8.43a	25.40±5.19c	150.61±1.48b

33	(Z,Z,Z)-9,12,15-Octadecatrienoic acid, methyl ester	Fatty	2095	0.87±0.01c	3.22±0.51c	87.62±7.08a	3.81±0.49c	24.86±0.52b
34	Acetic acid, 2-phenylethyl ester	Floral	1253	17.31±3.48d	93.79±1.28b	215.74±3.25a	20.56±2.48d	64.46±3.73c
35	Butanoic acid, 2-methyl-, hexyl ester	Green	1237	13.99±3.48c	24.91±1.09b	34.10±0.96a	4.97±0.46d	14.42±1.39c
36	5-ethyldihydro-2(3H)-Furanone	Fragrance of coconut	1049	26.71±1.20d	90.99±3.13c	137.99±1.95b	135.57±16.65b	222.48±12.53a
37	trans-2-Hexenyl Butyrate	Green,fruity	1196	44.12±3.16c	170.31±0.46b	453.78±7.70a	9.09±1.26d	38.40±3.13c
38	Butanoic acid, hexyl ester	Fruity	1193	17.48±1.29 c	54.39±4.24b	186.41±12.25a	7.48±0.82d	27.81±2.21c
39	Limonene	Lemon-like	1034	8.79± 1.87d	4.10±0.45d	20.05±5.05c	45.33±3.88a	35.66±2.84b
40	β-Ocimene	Citrus, berbaceous, sweet	1047	8.51±1.35d	439.26±25.73a	82.18±6.37b	28.94±2.06c	17.46±1.65c
41	2-pentyl-Furan	Fruity	989	10.47±1.59c	8.04±0.48c	19.22±1.43c	106.41±8.18b	217.15±18.14a
42	Coumarin	Tonka	1430	23.46±2.54b	26.56±2.59b	39.17±4.97a	26.42±4.65b	30.12±1.62b

*Odour descriptions were from the database (Flavornet; Perflavory) and the literatures (Flaig et al., 2020; Guo et al., 2021; Joshi & Gulati, 2015; Liao et al., 2020; Magagna et al., 2017; Sheibani et al., 2016)
