# Supplementary figures and images for: Effect of Mechanical Damage in Green-Making Process on Aroma of Rougui Tea
Source: Foods. 2024 Apr 25;13(9):1315. doi: 10.3390/foods13091315 (PMC11083345; doi:10.3390/foods13091315)

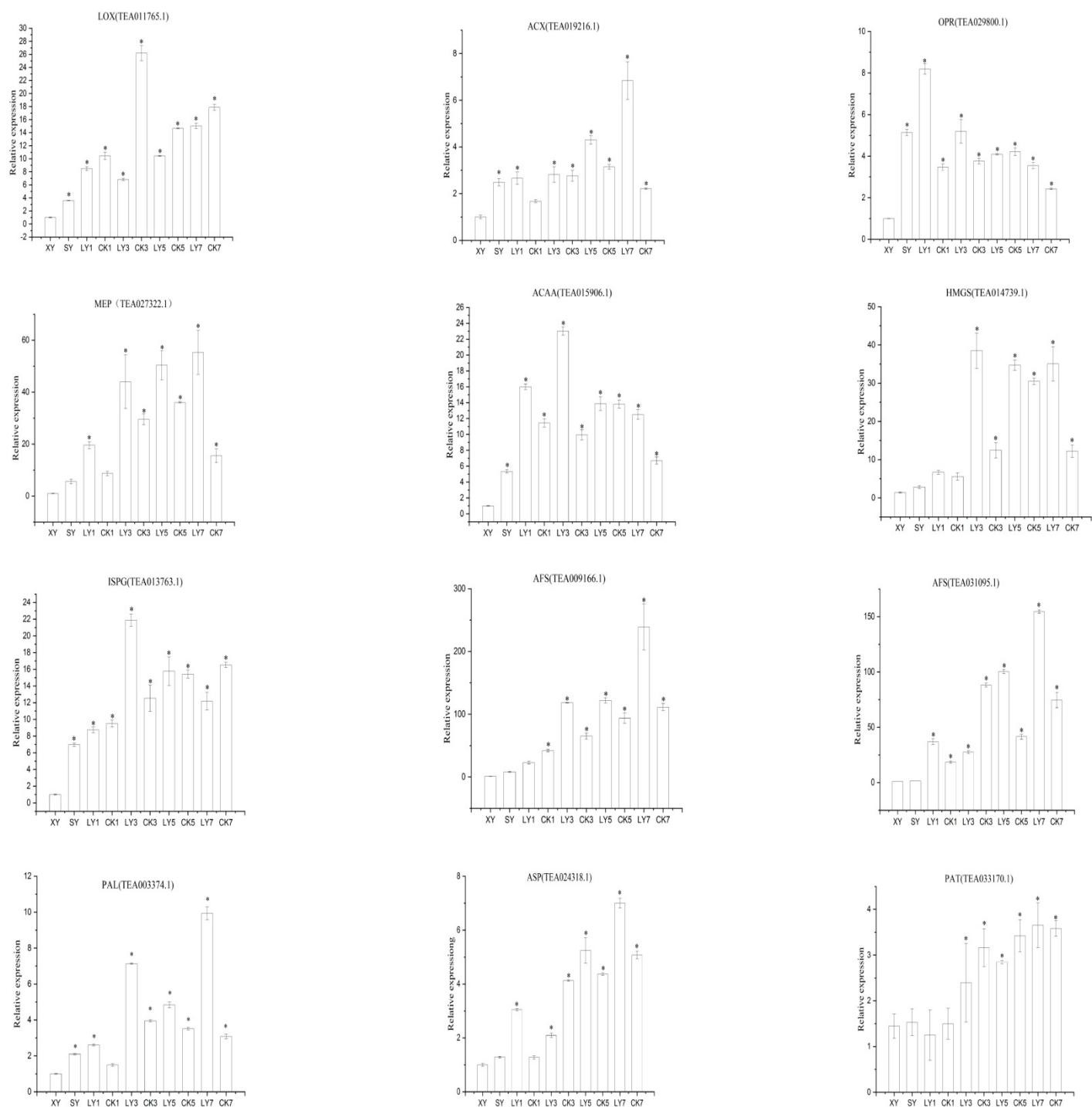

**Figure S1.** The expression level of related genes in different samples based on RT-PCR analysis.

Supplement: Supplementary file 1 [file foods-13-01315-s001.zip › Figure.S1.pdf]
